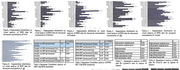# Whole Brain Echo‐planar Mr Spectroscopy For Metabolic Mapping In Alzheimer's Disease: ASimultaneous MR/PET Study

**DOI:** 10.1002/alz70856_098736

**Published:** 2025-12-24

**Authors:** Sandhya Mangalore

**Affiliations:** ^1^ National Institute of Mental Health and Neurosciences, Bengaluru, India

## Abstract

**Background:**

Echo planar MR Spectroscopy (EPSI), a promising diagnostic tool for AD, offeringinsights into the metabolic alterations associated with the disease.

**Method:**

In a prospective cross‐sectional observational study, 25 patients clinically diagnosed with ADand 10 healthy control underwent EPSI as part of their MRI protocol from July 2021 to July 2023. Weemployed EPSI MRI to assess the distribution (Cortex ID) and concentration of brain metabolites, correlating these findings with atrophy patterns on MRI and FDG ‐ PET.

**Result:**

EPSI analysis revealed a cerebral decrease in N‐acetylaspartate (NAA), particularly within the rightparietal inferior lobe (66.6%) and right occipital lobe (57.1%). Choline (Cho) showed significant decreasesnoted in the right parietal inferior lobe (50%) followed by right parietal superior lobe and temporal mediallobe (30%). Creatine (Cr) showed decreased level of concentration in right parietal inferior (60%) and rightoccipital lobe (40%). Glutamine/glutamate (Glx) showed significant decrease in the right precuneus (71.4%), aligning with neuronal dysfunction narratives in AD. Lactate (Lac) had increased concentration in leftparietal inferior lobe (57.1%) followed by right posterior cingulate gyrus (47.6%). Myo‐inositol (mI) levelsvaried, with increased concentrations in the right anterior cingulate gyrus (62%), suggesting glial activationin the AD pathology. Quantitative voxel‐based analysis demonstrated significant alterations in metaboliteratios, including NAA/Cr, Glx/Cr, Cho/Cr, mI/Cr, and Lactate/Cr, across various brain regions of AD patients. The decreased concentration of NAA across cerebral regions and the increased mI and lactate levelscontribute to supporting the role of EPSI in early diagnosis and in monitoring the effects of therapeuticinterventions.

**Conclusion:**

The integration of EPSI with MRI and FDG‐PET imaging provides a comprehensive approachto understanding the metabolic underpinnings of AD and paves the way for the development ofmetabolite‐based biomarkers for early detection and prognosis. Advantage of EPSI over other modalities is that it can be registered with MP_RAGE and hence Multi‐planar reconstruction can be done